# Identification of appropriate reference genes for RT-qPCR analysis in *Juglans regia* L.

**DOI:** 10.1371/journal.pone.0209424

**Published:** 2018-12-18

**Authors:** Li Zhou, Jianxin Niu, Shaowen Quan

**Affiliations:** 1 Department of Horticulture, College of Agriculture, Shihezi University, Shihezi, Xinjiang, P.R. China; 2 Xinjiang Production and Construction Corps Key Laboratory of Special Fruits and Vegetables Cultivation Physiology and Germplasm Resources Utilization, Shihezi, Xinjiang, P.R. China; Northwestern University Feinberg School of Medicine, UNITED STATES

## Abstract

Reverse transcription quantitative real-time PCR (RT-qPCR) is a popular adopted technique to detect gene expression, and the selection of appropriate reference genes is crucial for data normalization. In the present study, seven candidate reference genes were screened to evaluate their expression stability in various flower buds, leaf buds, tissues and cultivars of the English walnut (*Juglans regia* L.) based on four algorithms (geNorm, Normfinder, Bestkeeper and RefFinder). The results demonstrated that *TUA*, *EF1* and *TUB* were appropriate reference genes for flower buds at different stages of female flower buds differentiation; *TUB* and *18S rRNA* were best for leaf buds at different stages of female flower buds differentiation; *TUB* and *TUA* were suitable for different cultivars; and *ACT2*, *18S rRNA* and *GAPDH* were useful for different tissues. Moreover, the expression of *ACT* was not stable among different flower buds, leaf buds and cultivars. The stability of reference genes were confirmed through the analysis of the expression of *SPL18* gene. These results will contribute to a reliable normalization of gene expression in *J*. *regia*.

## Introduction

With high sensitivity, rapidity, specificity and reliability, reverse transcription quantitative real-time PCR (RT-qPCR) is a popular technique applied to detect relative gene expression [1, 2]. Nevertheless, the reliability of the data is influenced by the quantity and quality of the templates, and the efficiency of the reverse transcription reaction and amplification [3, 4]. Consequently, it is very important to assess gene expression levels with a stable reference gene. Numerous genes related to basic metabolism or cellular processes have been applied as reference genes in various fruit trees, including Chinese jujube [5, 6], peach [7, 8], litchi [9], grape [10], citrus [11], apple [12–14], pear [15, 16] and cherry [17]. Only one study has analyzed the reference genes stability for *Juglans sigillata* Dode [18].

The English walnut (*J*. *regia* L.) has abundant nutrition and commercial value, and is one of the most important nut fruit trees in the world. Flowering is an important stage for nut production. For most walnut trees, there is a long juvenile period of 8 to 10 years before first flowering. However, the *J*. *regia* cv. Xinxin 2, an early-seeding cultivar, has a short juvenile phase of 2 to 3 years, and few studies have explored the molecular mechanism of floral induction. Recently, we used high-throughput sequencing technology to detect the transcriptome profiles of flower buds and leaf buds before, during and after the critical period of female flower bud differentiation. The transcriptome sequence dataset provided abundant information for the selection of appropriate reference genes.

An ideal reference gene should be stably expressed not only in various tissues but also at all developmental stages. Furthermore, the reference gene should not be affected by experimental treatments [19]. However, an increasing number of studies indicated that expression of many classic reference genes varies among different tissues, genotypes and experimental treatments [4]. Hence, it is necessary to screen a suitable reference gene before RT-qPCR analysis.

In the present study, we screened seven candidate reference genes based on the transcriptome dataset of *J*. *regia* cv. Xinxin 2. Their expression stability among various flower buds, leaf buds, tissues and cultivars was assessed. Furthermore, the expression of one target gene (*SPL18*) was explored to validate the effectiveness of the selected reference genes.

## Materials and methods

### Plant materials

The plant samples in this study were collected from the southern part of Xinjiang Uyghur Autonomous Region, China. Flower buds and leaf buds were collected from *J*. *regia* cv. Xinxin 2 at different stages of female flower buds differentiation. Flower buds were collected at the critical period of female flower buds differentiation from the following four cultivars: Xinxin 2, Hetian, Wuhuo and Wen 185. Different tissues (leaves, leaf buds, branches and flower buds) were collected at the critical period of female flower buds differentiation from Xinxin 2. All samples were immediately frozen in liquid N and then stored at −70°C. For flower buds and leaf buds, three buds made a sample. Three samples were used for each period. (For each period, nine buds were used.). For different tissues, three samples were used. Eight periods of flower buds and five periods of leaf buds were considered to analysis the stability of the seven candidate reference genes.

### RNA isolation and cDNA synthesis

Total RNA was isolated from the flower buds, leaf buds, leaves and branches using a Plant RNA Extract Kit (Aidlab, Beijing, China). The quality of RNA was detected by NanoDrop 2000 (Thermo Scientific) and 1.1% agarose gel electrophoresis. For each sample, one microgram of total RNA was used to synthesize cDNA using a PrimeScript RT reagent Kit with gDNA Eraser (Takara, Dalian, China).

### Selection of candidate reference genes and primer design

Seven candidate reference genes, including actin (*ACT*), actin-related protein 2 (*ACT2*), elongation factor (*EF1*), α-Tublin (*TUA*), β-Tublin (*TUB*), glyceraldehyde-3-phosphatedehydrogenase (*GAPDH*), and 18S ribosomal RNA (*18S rRNA*) were selected based on their low variance of gene expression in the transcriptome dataset of *J*. *regia* cv. Xinxin 2. The primers were designed using Primer Premier 6 [20] and the detailed information were listed in S1 Table.

### RT-qPCR and statistical analysis

The RT-qPCR was conducted using a SYBR Green based PCR assay (Toyobo, Japan) on a Bio-Rad CFX96 real time PCR system. Each 10 μL PCR reaction mixture covered cDNA (1 μL), forward and reverse primer (0.4 μL), SYBR Green Mix (5 μL) and ddH_2_O (3.2 μL). The PCR procedure were 94°C for 30 s, followed by 40 cycles of 94°C for 15 s, 60°C for 15 s and 72°C for 30 s. A melting curve was performed to confirm the specificity of primers. Each reaction was carried out three times. In addition, standard curves were generated using a five-fold cDNA dilution series to calculate the amplification efficiency (E) and correction coefficients (R^2^) of the cand idate reference genes [2].

Quantification cycle (Cq) values were obtained and analyzed using three Microsoft Excel-based softwares, geNorm [21], Norm Finder [22], and BestKeeper [23]. The comprehensive ranking order of the candidate reference genes were obtained through an online-based program: RefFinder (http://150.216.56.64/referencegene.php?type=reference), which integrates the analysis of geNorm, Normfinder and Bestkeeper.

### Validation of candidate reference gene

To confirm the stability of reference genes, RT-qPCR was performed to detect the expression levels of squamosa promoter-binding-like protein 18 (*SPL18*) using the 2^-ΔΔCq^ method. Three of the most stable genes and the least stable gene were used to normalize the expression levels of *SPL18* in different flower buds and leaf buds at different stages of female flower buds differentiation (five periods were analyzed), different tissues and four cultivars. The primers of *SPL18* were 5’-AGCAGTGCAGCAGGTTCCATTC (forward) and 5’- GTCGTCGGTTGTGTCCATCAAGG (reverse).

## Results

### Primer specificity and PCR amplification efficiency

The specific primers for seven reference candidates were designed for RT-qPCR. The size of amplicons ranged from 98 to 166 bp. All primer pairs amplified a single PCR product of the expected size (S1 Fig). All the primers demonstrated a single peak melting curves (S2 Fig). The R^2^ of seven candidates ranged from 0.947 for *ACT2* to 0.997 for *EF1* and E varied from 97.8% to 111.1% for *TUB* and *ACT2*, respectively (S3 Fig).

### Expression profile of the reference genes

**Fig 1:**
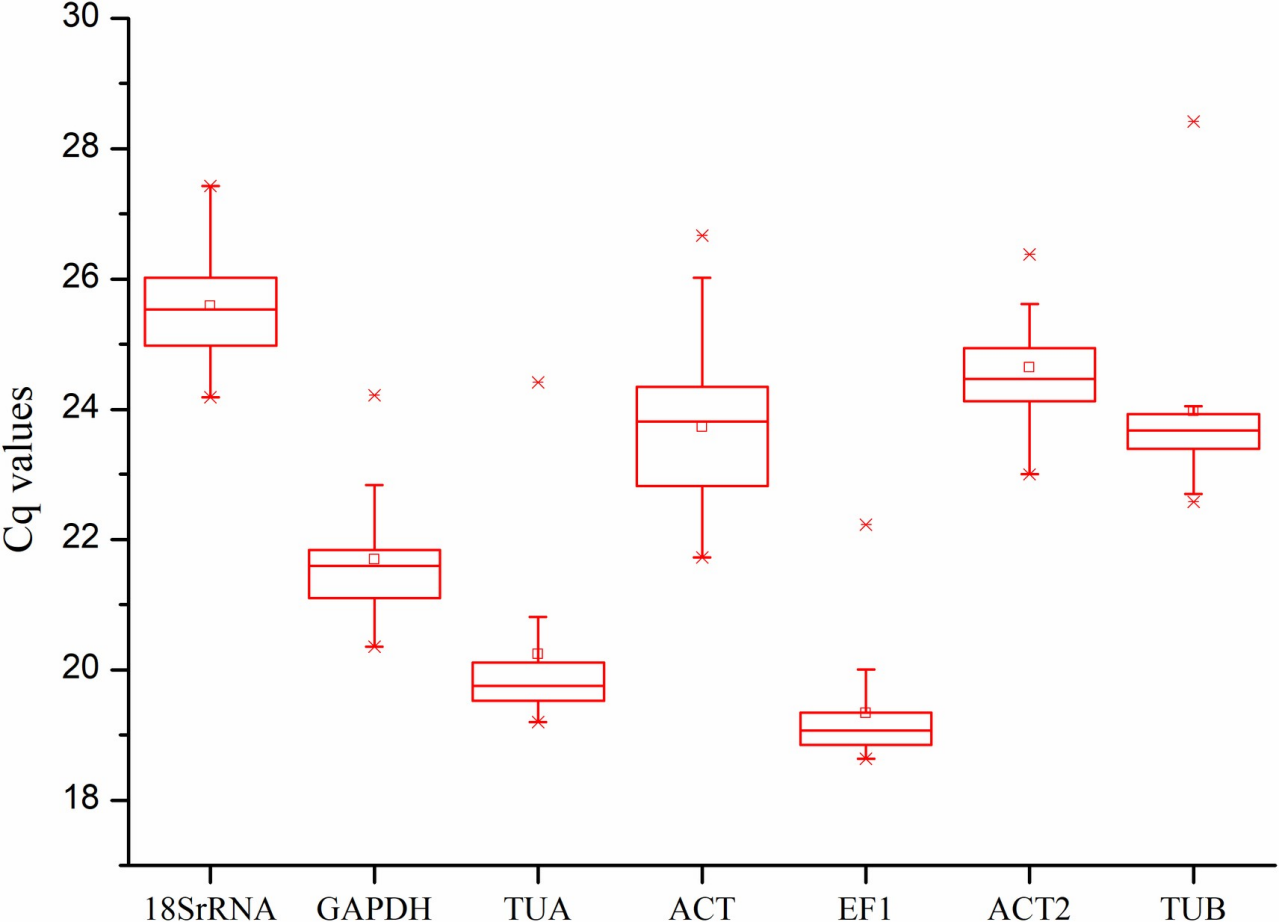
Expression levels (Cq values) of the seven candidate reference genes in all the tested samples. The lower and upper ends of box indicate the 1/4 and 3/4 quartiles. Whiskers indicate the maximum and minimum Cq values. The line in the box indicates the median and the small box indicates the mean Cq value. The star indicates the outlier.

The Cq values showed an overview of the expression level of seven candidate reference genes among the tested samples. The Cq values of the seven reference genes ranged from 18.63 (EF1) to 28.42 (TUB). The mean Cq values of the seven reference genes ranged from 19.34 to 25.58 (Fig 1, S2 Table). A high Cq value suggested a low gene expression level [24]. Among the seven candidate reference genes, *18S rRNA* and *ACT2* demonstrated low expression with high Cq values, whereas *TUA* and *EF1* showed high expression levels with low Cq values. The outliers suggested that no candidate reference genes had constant expression levels among all the tested samples. Therefore, it’s necessary to screen appropriate reference genes via statistical methods.

### Expression stability of candidate reference genes

#### geNorm analysis

**Fig 2:**
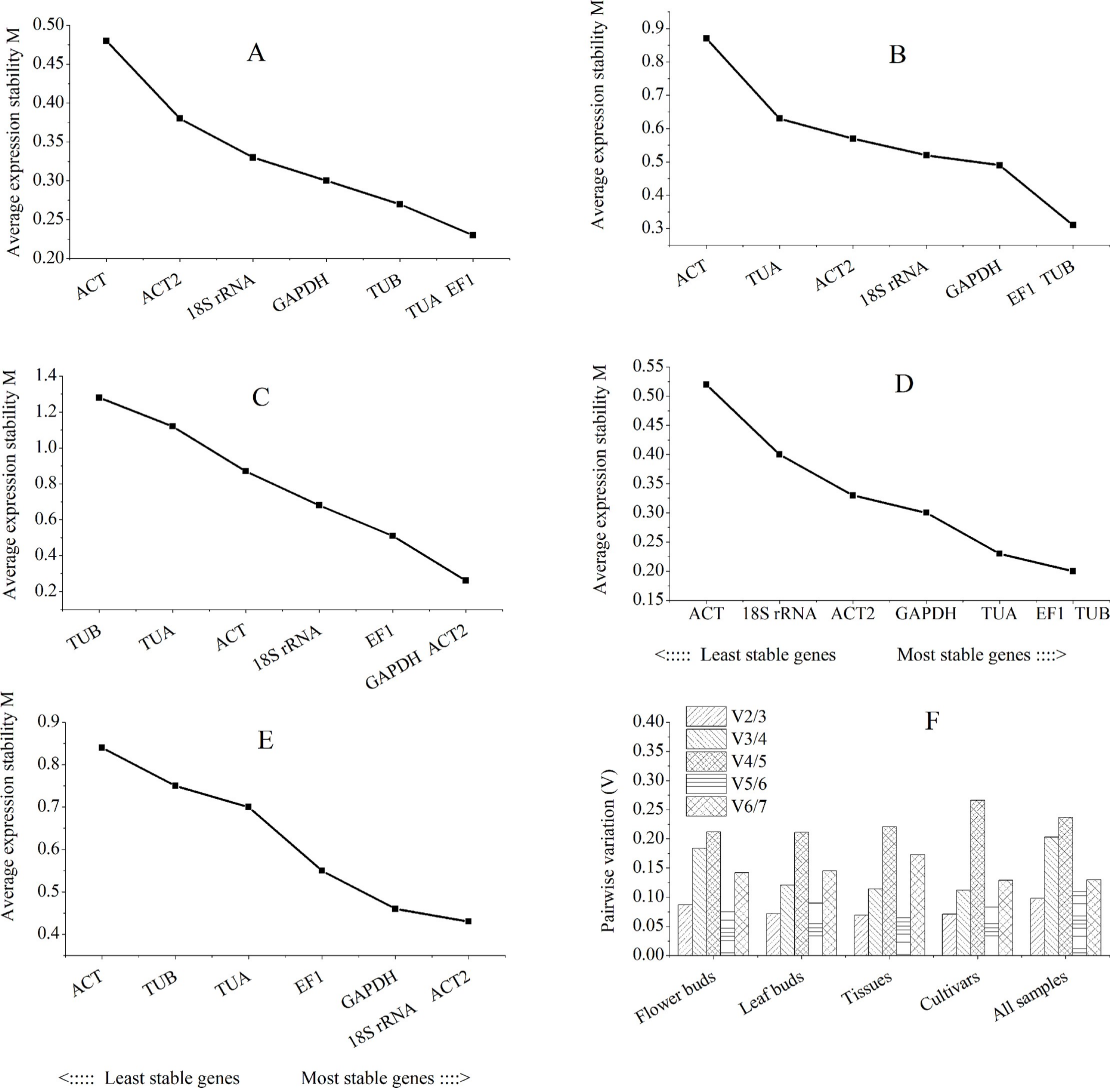
geNorm ranking of the seven candidate reference genes and pairwise variation analysis to select the optimal number of reference genes. A: flower buds, B: leaf buds, C: different tissues, D: four cultivars, E: all samples. F: pairwise variation.

GeNorm algorithm determines the ranking of candidate reference genes by calculating the expression stability measure (M). The M value of the gene is negatively related to its stability, and 1.5 is set as the threshold of M value [21]. As showed in Fig 2, M-values of all the seven candidate reference genes were below 1.5. The stability of the seven candidate reference genes varied across the tested samples. Among the various flower buds, *TUA* and *EF1* were the most stable genes with a same M-value of 0.23 (Fig 2A). *EF1* and *TUB* were the most stable genes with a same M-value among the different leaf buds (Fig 2B) and the four cultivars (Fig 2D). For different tissues, *GAPDH* and *ACT2* were the most stable genes with a same M-value of 0.26 (Fig 2C). When all the samples were analyzed, 18S rRNA and ACT2 were the most stable genes (Fig 2E). ACT was found to be the least stable gene among the various flower buds, leaf buds, different cultivars and all the test samples (Fig 2A, 2B, 2D and 2E).

The geNorm also generated the pairwise variation (V_n/n+1_), which can help to select the optimal number of references. If the value of V_n/n+1_ was less than 0.15, there is no necessary to apply an additional reference gene (Vandesompele *et al*., 2002). For various flower buds, the value of V_2/3_ was 0.087 (Fig 2F), suggesting that two reference genes, *EF1* and *TUA*, were needed for normalization. For leaf buds at different flowering stages, the variation value was 0.121 at V_3/4_, so three reference genes, *TUB*, *EF1* and *GAPDH*, were required for normalization. The value of V_2/3_ was 0.075 in the cultivars group, suggesting that *EF1* and *TUB* were needed for normalization.

#### NormFinder analysis

The results of NormFinder analysis were shown in Table 1. For various flower buds, *TUA* (0.085), *TUB* (0.145) and *EF1* (0.193) ranked the most stable genes, which was similar to the results generated from geNorm. Similarly, *ACT* was the least stable gene among the various flower buds, leaf buds, cultivars and all the tested samples calculated by NormFinder, which was consistent with the results of geNorm. For various leaf buds, tissues and cultivars, the most stable genes got from NormFinder were different from the results of geNorm. Specifically, *18S rRNA* (0.155) and *TUB* (0.235) for various leaf buds; *ACT* (0.115) and 18S rRNA (0.199) for different tissues; *GAPDH* (0.097), *TUA* (0.167) and *TUB* (0.206) for different cultivars were ranked the most stable by NormFinder.

**Table 1:** The stability of candidate reference genes based on Normfinder analysis.

Rank	Flower buds		Leaf buds		Different tissues		Different cultivars		All samples	
	Gene	Stability value	Gene	Stability value	Gene	Stability value	Gene	Stability value	Gene	Stability value
1	*TUA*	0.085	*18S rRNA*	0.155	*ACT*	0.115	*GAPDH*	0.097	*18S rRNA*	0.169
2	*TUB*	0.145	*TUB*	0.235	*18S rRNA*	0.199	*TUA*	0.167	*ACT2*	0.290
3	*EF1*	0.193	*ACT2*	0.272	*ACT2*	0.494	*TUB*	0.206	*GAPDH*	0.361
4	*GAPDH*	0.200	*GAPDH*	0.370	*GAPDH*	0.607	*ACT2*	0.221	*TUA*	0.443
5	*18S rRNA*	0.211	*EF1*	0.372	*TUA*	0.843	*EF1*	0.245	*EF1*	0.473
6	*ACT2*	0.269	*TUA*	0.428	*EF1*	0.976	*18S rRNA*	0.256	*TUB*	0.491
7	*ACT*	0.469	*ACT*	0.975	*TUB*	1.121	*ACT*	0.529	*ACT*	0.611

#### BestKeeper analysis

Bestkeeper was applied to evaluate the stability of candidate references according to the standard deviation (SD) and the coefficient of variation (CV) of Cq values. SD values less than 1 were acceptable. A lower SD and CV (CV ± SD) value indicated a more stable gene. For various flower buds, the values of SD for seven candidate reference genes were below 1 (Table 2). *EF1* (CV ± SD = 1.60% ± 0.31) were ranked the best stability, followed by *TUA* (CV ± SD = 1.69% ± 0.33) and *TUB* (CV ± SD = 1.73% ± 0.41). This result was similar with that got from geNorm and NormFinder. However, in other experimental groups, the results of Bestkeeper analysis were different from those got from geNorm and NormFinder. For various leaf buds, *18S rRNA* was the most stable (CV ± SD = 2.89% ± 0.76), followed by *ACT2* (CV ± SD = 3.10% ±0.79) and *GAPDH* (CV ± SD = 3.60% ± 0.82). All other genes, with SD values bigger than 1, were not acceptable. For different tissues, *EF1* had the lowest CV ± SD values (1.87%±0.36), followed by *ACT2*, *GAPDH* and *18S rRNA*, whereas *ACT*, *TUA* and *TUB* had SD values bigger than 1. For different cultivars, all the genes had SD values less than 1, and *TUB* were ranked the most stable, followed by *TUA* and *EF1*. For all samples, *EF1* was the most stable gene.

**Table 2:** The stability of candidate reference genes based on Bestkeeper analysis.

	Flower buds			Leaf buds			Different tissues			Different cultivars			All samples		
Rank	Gene	SD	CV (%)	Gene	SD	CV (%)	Gene	SD	CV (%)	Gene	SD	CV (%)	Gene	SD	CV (%)
1	*EF1*	0.31	1.60	*18srRNA*	0.76	2.89	*EF1*	0.36	1.87	*TUB*	0.11	0.49	*EF1*	0.55	2.86
2	*TUA*	0.33	1.69	*ACT2*	0.79	3.10	*ACT2*	0.52	2.08	*TUA*	0.13	0.68	*ACT2*	0.62	2.53
3	*TUB*	0.41	1.73	*GAPDH*	0.82	3.60	*GAPDH*	0.54	2.45	*EF1*	0.15	0.80	*GAPDH*	0.65	3.01
4	*ACT2*	0.43	1.78	*TUB*	1.22	5.01	*18S rRNA*	0.88	3.40	*GAPDH*	0.20	0.94	*18srRNA*	0.68	2.66
5	*GAPDH*	0.49	2.33	*EF1*	1.23	6.15	*ACT*	1.07	4.49	*ACT2*	0.23	0.93	*TUB*	0.82	3.41
6	*18S rRNA*	0.54	2.14	*ACT*	1.26	5.26	*TUA*	1.62	7.63	*18S rRNA*	0.37	1.45	*TUA*	0.87	4.30
7	*ACT*	0.65	2.76	*TUA*	1.28	6.16	*TUB*	1.82	7.34	*ACT*	0.62	2.65	*ACT*	0.88	3.69

#### Comprehensive ranking of the candidate reference genes by RefFinder

The comprehensive ranking order of the candidate reference genes were obtained through RefFinder, which integrated the methods of geNorm, NormFinder and Bestkeeper. As shown in Table 3, *TUA*, *EF1* and *TUB* were the most three stable genes in flower buds. *18S rRNA* and *TUB* expressed most stably in leaf buds. For different tissues, *ACT2*, *18S rRNA* and *GAPDH* were the most suitable reference genes. For different cultivars, *TUB*, *TUA* and *GAPDH* were identified as the most stable genes. Among all the samples, *18S rRNA*, *ACT2* and *GAPDH* were expressed most stably. The expression of *ACT* was unstable in flower buds, leaf buds and different cultivars. The expression of *TUB* was unstable across different tissues.

**Table 3:** Expression stability ranking of the candidate reference genes based on RefFinder.

Method	1	2		3	4	5	6	7
**Ranking Order In Flower Buds (Better—Good—Average)**								
BestKeeper	*EF1*	*TUA*		*TUB*	*ACT2*	*GAPDH*	*18S rRNA*	*ACT*
Normfinder	*TUA*	*TUB*		*EF1*	*GAPDH*	*18S rRNA*	*ACT2*	*ACT*
Genorm	*TUA | EF1*			*TUB*	*GAPDH*	*18S rRNA*	*ACT2*	*ACT*
RefFinder	*TUA*	*EF1*		*TUB*	*GAPDH*	*18S rRNA*	*ACT2*	*ACT*
**Ranking Order In Leaf Buds (Better—Good—Average)**								
BestKeeper	*18S rRNA*	*ACT2*		*GAPDH*	*TUB*	*EF1*	*ACT*	*TUA*
Normfinder	*18S rRNA*	*TUB*		*ACT2*	*GAPDH*	*EF1*	*TUA*	*ACT*
Genorm	*EF1 | TUB*			*GAPDH*	*18S rRNA*	*ACT2*	*TUA*	*ACT*
RefFinder	*18S rRNA*	*TUB*		*ACT2*	*EF1*	*GAPDH*	*TUA*	*ACT*
**Ranking Order In Differ Tissues (Better—Good—Average)**								
BestKeeper	*EF1*	*ACT2*		*GAPDH*	*18S rRNA*	*ACT*	*TUA*	*TUB*
Normfinder	*ACT*	*18S rRNA*		*ACT2*	*GAPDH*	*TUA*	*EF1*	*TUB*
Genorm	*GAPDH | ACT2*			*EF1*	*18S rRNA*	*ACT*	*TUA*	*TUB*
RefFinder	*ACT2*	*18S rRNA*		*GAPDH*	*ACT*	*EF1*	*TUA*	*TUB*
**Ranking Order Indifferent Cultivars (Better—Good—Average)**								
BestKeeper	*TUB*	*TUA*		*EF1*	*GAPDH*	*ACT2*	*18S rRNA*	*ACT*
Normfinder	*GAPDH*	*TUA*		*TUB*	*ACT2*	*EF1*	*18S rRNA*	*ACT*
Genorm	*EF1 | TUB*			*TUA*	*GAPDH*	*ACT2*	*18S rRNA*	*ACT*
RefFinder	*TUB*	*TUA*		*GAPDH*	*EF1*	*ACT2*	*18S rRNA*	*ACT*
**Ranking Order In All Samples (Better—Good—Average)**								
BestKeeper	*EF1*	*ACT2*		*GAPDH*	*18S rRNA*	*TUB*	*TUA*	*ACT*
Normfinder	*18S rRNA*	*ACT2*		*GAPDH*	*TUA*	*EF1*	*TUB*	*ACT*
Genorm	*18S rRNA | ACT2*			*GAPDH*	*EF1*	*TUA*	*TUB*	*ACT*
RefFinder	*18S rRNA*	*ACT2*		*GAPDH*	*EF1*	*TUA*	*TUB*	*ACT*

### Validation of selected reference genes

**Fig 3:**
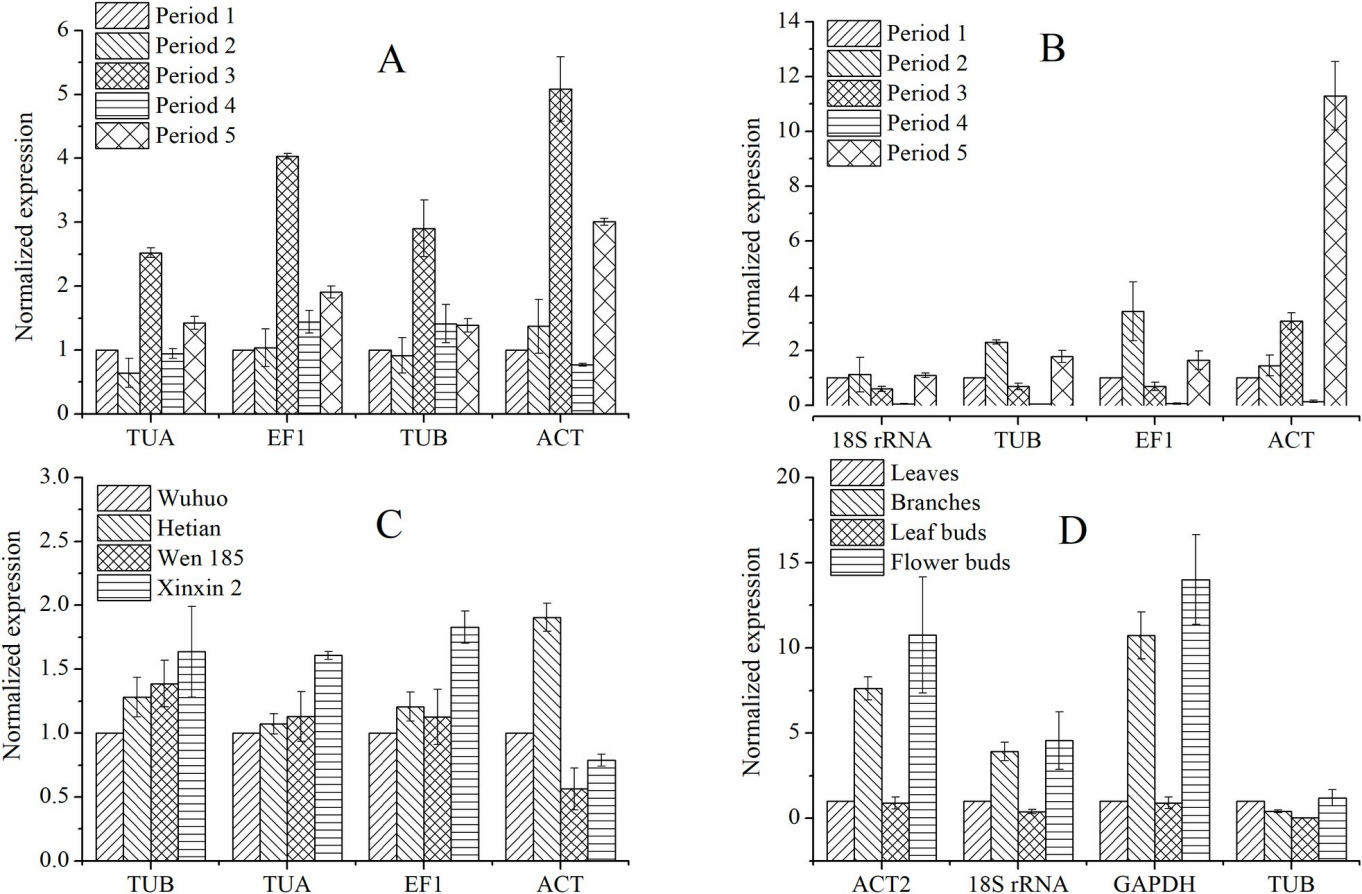
Relative expression of *SPL18* based on candidate reference genes. A: flower buds. B: leaf buds. C: different cultivars. D: different tissues. The relative expression levels were depicted as mean+ SD (standard deviation) calculated from three technical replicates.

The expression levels of *SPL18* were assessed in various flower buds, leaf buds, tissues and cultivars to confirm the stability of the selected reference genes. Three of the most stable reference genes and the most unstable gene were applied for data normalization. The expression levels of *SPL18* were similar in different flower buds under the normalization of *TUA*, *EF1* and *TUB* (Fig 3A). Similar results were obtained when *18S rRNA*, *TUB* and *EF1* were used as reference genes. However, when the least stable gene (ACT) was used as the reference gene, the expression of *SPL18* was considerably biased at Period 5 (Fig 3B). Analysis of four cultivars suggested that the expression levels of *SPL18* were consistent when *TUB* and *TUA* were used for normalization, while there was a slightly difference when *EF1* was used as reference gene. However, there was a significant difference when *ACT* was applied for normalization (Fig 3C). For different tissues, the expression trends of *SPL18* were consistent when *ACT2*, *18S rRNA* and *GAPDH* were used for normalization. *TUB* was not suitable for normalization among different tissues (Fig 3D).

## Discussion

Different normalization approaches can change the calculation of P-values and fold changes of a large number of genes depending on the normalization method applied [25]. RT-qPCR is considered as the most appropriate method for gene expression due to its high sensitivity, rapidity, specificity and reliability [1, 2]. An ideal reference gene is assumed to have constant expression levels among different samples and under different experimental conditions. However, there is no universal reference gene. The expression of putative reference genes varies across different tissues, genotypes and various experimental conditions [26]. The expression levels of target genes were evaluated according to the expression of reference genes, however, an unstable reference gene can result in inaccurate evaluation of target gene expression. For example, the expression of *FaWRKY1* in roots of tall fescue under salinity and drought stress peaked at 3 h when the most stable reference genes (*TUB* and *SAND*) were used. However, the expression of *FaWRKY1* exhibited fluctuations and failed to achieve a consistent pattern when the least stable gene (*EF1α*) was used [27]. As reported in the previous study, the expression patterns of miR159 for leaf, stem and root were consistent when the most stable reference genes, *EF-1α*, *Ubiquitin* and *GAPDH*, were used for data normalization. However, when the least stably expressed reference genes, *Actin* and *18S rRNA*, were used for data normalization, the expression of miR159 was considerably biased. This result indicated that the least stable reference genes, *Actin* and *18S rRNA*, failed to standardize the expression data effectively [28]. In this study, the expression level and stability of seven candidate reference genes were evaluated by RT-qPCR using geNorm, Normfinder and Bestkeeper. As a result, no single reference gene was consistently expressed across all the samples tested due to the different statistical algorithms of the programs [29]. For example, *EF1*, *TUA* and *TUB* were identified as the most stable genes in various flower buds and different cultivars by geNorm, Normfinder and Bestkeeper. Similar to our results, *EF1* and *TUB* were steadily expressed during flower development in other plants [30, 31]. However, *EF1* and *TUA* in various leaf buds; *TUA*, *EF1* and *TUB* in different tissues were not stable by Normfinder (Table 2). *TUB* and *TUA* were also identified to be unstable reference genes in peach [7]. Similarly, *EF1* was not stably expressed in grapevine [10]. A previous study indicated that *β-ACT2* was not an appropriate reference gene in *Juglans sigillata [18]*. In the present study, *ACT* was also identified as the least stable in various flower buds and leaf buds and different cultivars by the three programs (Fig 2, Tables 1 and 2), whereas it was ranked first in different tissues by Normfinder (Table 2). These results may be due to different species and tissues that were assessed as reliable reference genes that were highly specific to an individual experimental condition. Furthermore, we applied RefFinder to get a comprehensive ranking order of the seven candidate reference genes based on the geometric mean of every reference gene evaluated through delta Cq, geNorm, Normfinder and Bestkeeper [19, 32, 33]. To confirm the stability of selected reference genes, the expression levels of *SPL18* in different flower buds, leaf buds, tissues and four cultivars were detected. The results revealed that *SPL18* were consistently expressed under the normalization of the most stable reference genes (Fig 3).

## Conclusion

In conclusion, the stability of seven candidate reference genes for RT-qPCR data normalization was evaluated by geNorm, Normfinder and Bestkeeper, and the comprehensive ranking orders were obtained by RefFinder. The results demonstrated that *TUA*, *EF1* and *TUB* for flower buds at different stages of female flower buds differentiation, *18S rRNA* and *TUB* for leaf buds at different stages of female flower buds differentiation, *TUB* and *TUA* for different cultivars, *ACT2*, *18S rRNA* and *GAPDH* for different tissues were considered to be the suitable reference genes. Moreover, *ACT* was not a suitable reference gene for different flower buds, leaf buds and cultivars. *TUB* expression was unstable among different tissues. Additionally, the expression level of *SPL18* was analyzed to validate the stability of selected reference genes. Our results will contribute to a reliable normalization of RT-qPCR data for gene expression in *J*. *regia*.

## Supporting information

S1 Fig Agarose gel (1.2%) electrophoresis shown amplification of seven candidate reference genes with a single PCR product of expected size. (TIF)

S2 Fig Melting curves of seven candidate reference genes in RT-qPCR. (TIF)

S3 Fig Standard curves of seven candidate reference genes in RT-qPCR. (TIF)

S1 Table Primer sequences and amplification characteristics of seven candidate reference genes for RT-qPCR analysis. (XLSX)

S2 Table The Cq values of seven candidate reference genes. (DOCX)
