# Supplementary figures and images for: Identification of appropriate reference genes for RT-qPCR analysis in Juglans regia L
Source: PLoS One. 2018 Dec 18;13(12):e0209424. doi: 10.1371/journal.pone.0209424 (PMC6298729; doi:10.1371/journal.pone.0209424)

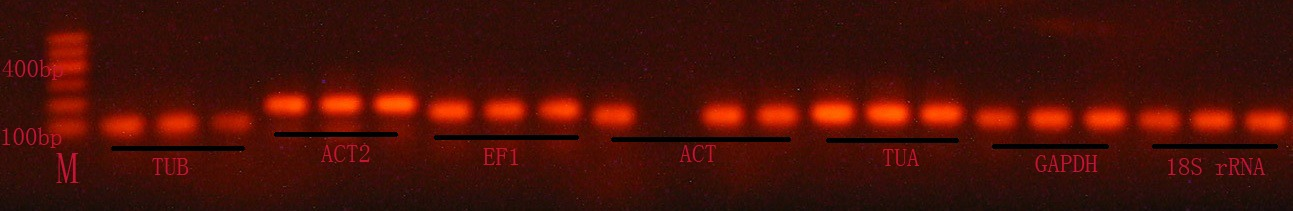

Supplement: S1 Fig — (TIF) [file pone.0209424.s001.tif]

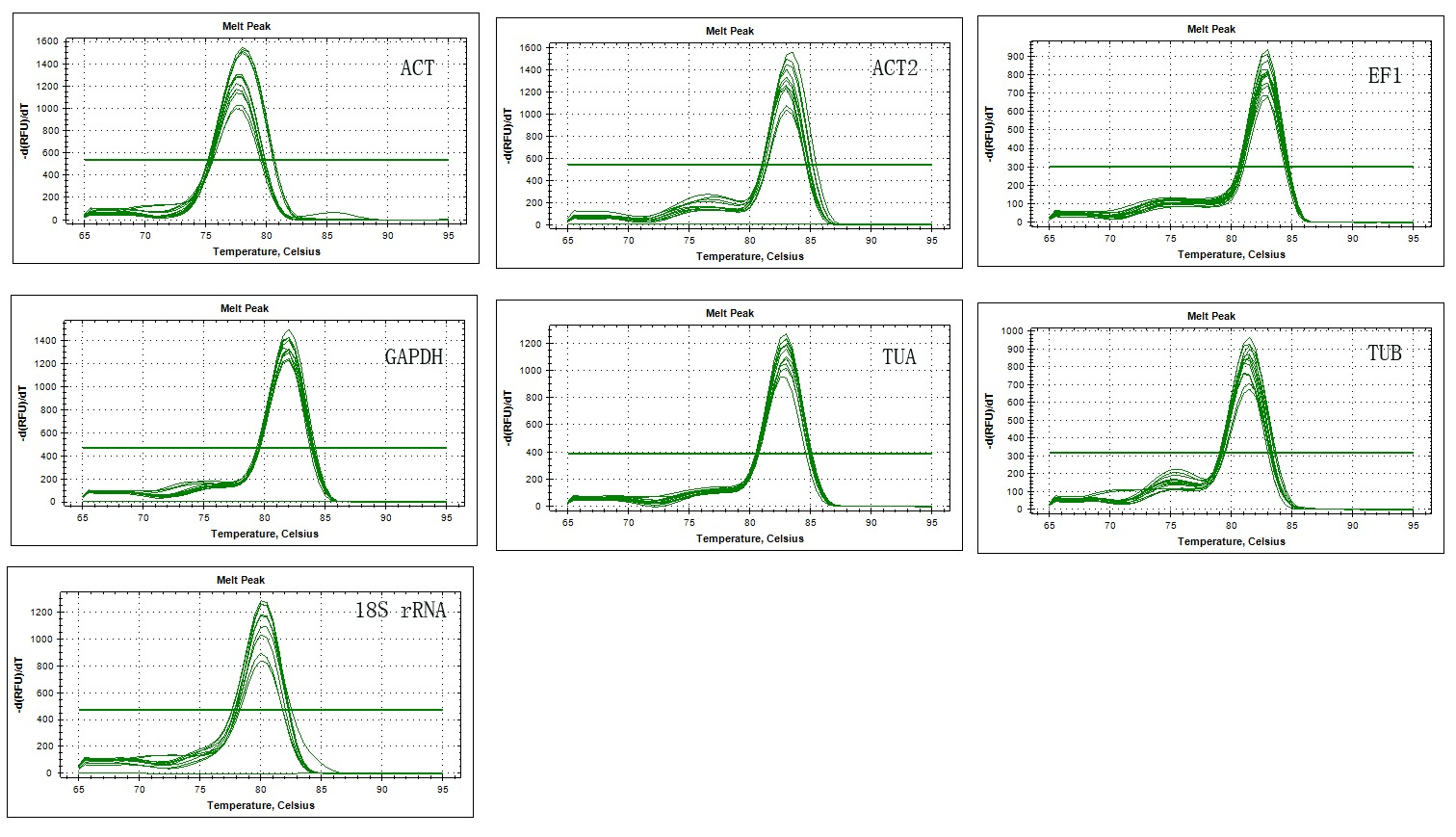

Supplement: S2 Fig — (TIF) [file pone.0209424.s002.tif]

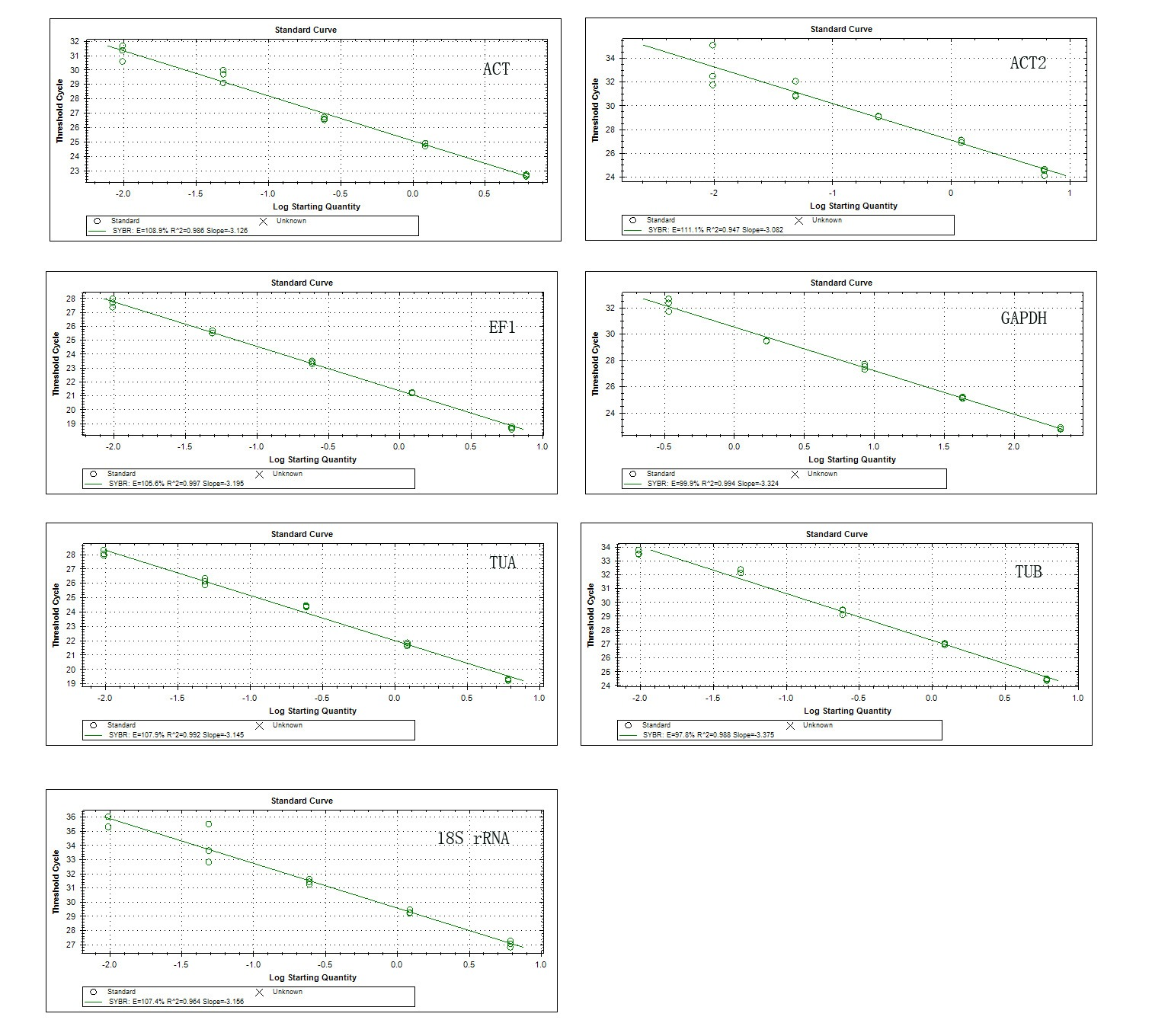

Supplement: S3 Fig — (TIF) [file pone.0209424.s003.tif]
