# Supplementary material for: Identification of appropriate reference genes for RT-qPCR analysis in Juglans regia L
Source: PLoS One. 2018 Dec 18;13(12):e0209424. doi: 10.1371/journal.pone.0209424 (PMC6298729; doi:10.1371/journal.pone.0209424)
Supplement: S2 Table — (DOCX) [file pone.0209424.s005.docx]

**S2 Table.** The Cq values of seven candidate reference genes.

|  | 18S rRNA | GAPDH | TUA | ACT | EF1 | ACT2 | TUB |
| --- | --- | --- | --- | --- | --- | --- | --- |
| Flower buds | 26.02 | 21.80 | 20.44 | 23.82 | 19.30 | 25.36 | 23.83 |
|  | 25.38 | 21.69 | 20.11 | 24.60 | 19.69 | 24.35 | 24.02 |
|  | 25.70 | 21.44 | 20.07 | 24.46 | 19.61 | 24.12 | 23.65 |
|  | 25.46 | 21.69 | 19.82 | 24.12 | 19.07 | 24.22 | 23.59 |
|  | 24.98 | 20.60 | 19.49 | 24.01 | 18.93 | 24.43 | 23.57 |
|  | 24.55 | 21.10 | 19.73 | 22.82 | 19.19 | 24.05 | 23.68 |
|  | 24.18 | 20.35 | 19.35 | 23.82 | 18.63 | 23.74 | 22.70 |
|  | 24.55 | 20.63 | 19.20 | 21.97 | 18.70 | 23.00 | 22.58 |
| Leaf buds | 25.69 | 21.84 | 19.99 | 21.72 | 18.79 | 24.57 | 23.38 |
|  | 27.42 | 24.22 | 22.17 | 23.94 | 22.23 | 26.28 | 26.30 |
|  | 26.04 | 22.83 | 19.29 | 24.45 | 19.34 | 24.57 | 23.92 |
|  | 25.53 | 21.59 | 19.76 | 23.13 | 18.92 | 24.94 | 22.98 |
|  | 27.26 | 23.21 | 22.51 | 26.67 | 20.94 | 26.38 | 25.66 |
| Different tissues | 25.53 | 21.59 | 19.76 | 23.13 | 18.92 | 24.94 | 22.98 |
|  | 26.24 | 22.59 | 20.81 | 23.54 | 20.01 | 25.46 | 24.05 |
|  | 27.36 | 22.25 | 24.41 | 26.02 | 19.03 | 25.61 | 28.42 |
|  | 24.55 | 21.10 | 19.73 | 22.82 | 19.19 | 24.05 | 23.68 |
| Different cultivars | 25.12 | 21.11 | 19.53 | 22.62 | 18.80 | 24.72 | 23.78 |
|  | 25.64 | 21.68 | 19.73 | 23.56 | 18.72 | 24.47 | 23.39 |
|  | 25.49 | 21.23 | 19.40 | 24.35 | 18.85 | 24.23 | 23.60 |
|  | 24.55 | 21.10 | 19.73 | 22.82 | 19.19 | 24.05 | 23.68 |
